# Frontal Polymerization of Epoxy Resins: Kinetic Modeling, Rate Regulation and Curing Process Simulation for Space Manufacturing Applications

**DOI:** 10.3390/polym17050680

**Published:** 2025-03-04

**Authors:** Haisheng Wu, Yizhuo Gu, Xinyu Liu, Chaobo Xin

**Affiliations:** 1 School of Materials Science and Engineering, Beihang University, Beijing 100191, China; whser@163.com (H.W.); benniegu@buaa.edu.cn (Y.G.); 2 Beijing Spacecrafts, Beijing 100094, China; 3 Langfang Feize Composites Technology Co., Ltd., Langfang 065000, China; chaobo.xin@fztec.net

**Keywords:** frontal polymerization, epoxy resin, curing mechanism, curing simulation

## Abstract

Frontal polymerization (FP) technology has attracted significant attention as an efficient, low-energy curing method for thermosetting resins. By enabling self-sustaining polymerization reactions, FP significantly reduces curing time and minimizes external energy dependence, making it ideal for in-orbit manufacturing applications. In contrast to traditional curing methods, which are limited by high energy consumption and low efficiency, FP offers a more efficient and flexible alternative. Nonetheless, the FP process is sensitive to material composition, processing and environmental factors, requiring systematic studies to enhance performance. This work focuses on reaction mechanisms, curing kinetics and processing factors of a self-developed FP epoxy resin system. The revealed curing mechanism and kinetics reveals a high initiation energy barrier and rapid curing characteristics, showing appropriate reaction inertness before initiation and stable reaction without continuous external energy input. The influences of initiator concentration and epoxy resin type on polymerization rate and the properties of cured resin were examined. Additionally, a curing simulation method validated by the experiment were employed to analyze the effects of mold material, resin cross-sectional area, initial temperature and environmental conditions on polymerization behavior. The results provide valuable insights for optimizing FP, advancing the understanding of the curing process and improving resin performance in space-based applications.

## 1. Introduction

The concept of in situ utilization of space resources was proposed even before humanity’s initial landing on the Moon, with the primary objective being the in situ production of propellants and life support consumables, primarily oxygen and water. At that time, manufacturing items in space was viewed as a highly futuristic goal [1]. However, recent rapid advancements in information technology, materials science, automation and rapid prototyping—particularly breakthroughs in additive manufacturing—have significantly progressed space manufacturing technologies [2,3,4]. Despite these advancements, current additive manufacturing technologies are predominantly confined to metals, thermoplastic resins and their composites reinforced by short fibers. The fabrication and processing of large-scale components require substantial amounts of high-density energy, while the manufacturing processes generates considerable waste heat, necessitating additional specialized thermal management. These factors result in low energy utilization efficiency, which severely limits the efficiency, scale and throughput of in-orbit manufacturing [5,6,7,8].

Frontal polymerization (FP) is an innovative in situ curing method for thermosetting resins. It relies on exothermic heat generated by the curing reaction itself as driving force, allowing the reaction zone to propagate continuously, ultimately converting all monomers into polymers [9,10,11]. The first successful demonstration of frontal polymerization was reported in 1972 by Russian scientists [12], who used methyl methacrylate and benzoyl peroxide as monomer and thermal initiator, respectively. However, this system faced challenges in maintaining a stable and continuous polymerization front due to heat loss [13]. Recent studies have explored the application of ring-opening metathesis polymerization (ROMP) of dicyclopentadiene (DCPD) in frontal polymerization. Notably, in 2018, Robertson et al. [14] introduced 3D printing technology of FP using pure DCPD. This method employed a second-generation Grubbs catalyst (Grubbs type II, GC2) to in situ cure unsupported and complex structures of DCPD polymers. Nevertheless, the polymerization rate of DCPD under the influence of GC2 is excessively rapid, posing significant challenges in reaction control and often necessitating specific inhibitors to extend the processing window for DCPD [15]. Moreover, the application scenarios for pure poly (DCPD) (pDCPD) are limited, which necessitates copolymerization modifications to enhance the specialized properties of DCPD. These challenges contribute to the relatively high cost and complexity-associated ROMP systems [16,17].

Epoxy resin continues to be the most prevalent matrix in composite materials due to its excellent mechanical properties, radiation resistance and chemical stability. When reinforced with fibers, epoxy resin serves effectively as a structural material. The integration of epoxy resin into frontal polymerization processes presents considerable potential. In comparison to ROMP, ring-opening polymerization of epoxy resin exhibits a lower heat generation rate, making epoxy resin-based frontal polymerization systems more robust than those using ROMP systems without inhibitors [9]. Currently, common initiators for frontal polymerization of epoxy resins include boron trifluoride (BF_3_)-amine complexes and photoacid generators (PAG). Scognamillo et al. [18] reported the cationic frontal polymerization of epoxy resins using trimethylolpropane triglycidyl ether (TMPTGE) and BF_3_-amine complex initiators. Initially, under thermal conditions, the BF_3_-amine complex quickly converts into a highly acidic active species, H^+^BF_4_^−^, initiating the polymerization. Subsequently, H^+^BF_4_^−^ reacts with TMPTGE to form a complex with a cationic active center, thereby triggering the etherification reaction between epoxy groups and promoting polymer chain growth, denoting the propagation stage. Finally, the active species terminate the reaction through ion pair rearrangement or the release of H^+^, leading to the formation of the cured resin. Zhou et al. [19] used boron trifluoride monoethylamine (BF_3_-MEA) as an initiator to successfully achieve the frontal polymerization of 3,4-epoxycyclohexylmethyl-3′,4′-epoxycyclohexane carboxylate (E221). They observed that as the initiator concentration increased, both the polymerization rate (*V_f_*) and the maximum temperature (*T*_max_) correspondingly increased. However, BF_3_-amine complexes are hygroscopic and prone to losing their activity, posing storage challenges. Additionally, they are applicable only to high-activity epoxy resins, which restricts their applicability and potential use cases.

Radical-induced cationic frontal polymerization (RICFP) represents an innovative curing mechanism for epoxy resins that employs photoacid generators (PAGs) to initiate cationic polymerization, resulting in ring opening and subsequent polymerization of the epoxy resin. The RICFP mechanism was first proposed by Mariani et al. [20], who utilized the heat released during the polymerization of epoxy monomers to promote the decomposition of active free radical initiators within the system. These free radicals induce the cleavage of PAGs, generating superacids that further initiate the ring opening of epoxy groups, thereby triggering the frontal polymerization process. Bomze et al. [21,22] conducted fundamental research on RICFP for various epoxy resins that contain unstable C—C compounds. They used 1,1,2,2-tetraphenyl ethanediol (TPED) as a thermal free radical initiator, in conjunction with diaryl iodonium salts, successfully achieving a self-sustaining frontal curing reaction based on bisphenol A diglycidyl ether (DGEBA) epoxy resin under localized thermal or photoinitiation. However, despite the development of corresponding epoxy resin RICFP systems, the curing process is susceptible to variations in curing rate and temperature gradients, which might lead to uneven curing and localized defects. Furthermore, the mechanisms governing the control of polymerization rates in the frontal polymerization of epoxy resin systems remain poorly understood.

While RICFP systems for epoxy resins have demonstrated great potential, some challenges, such as inconsistent polymerization rate and large temperature gradient, lead to uneven curing. These issues stem from insufficient understanding of the coupled radical-cationic reaction mechanism and their kinetic dependencies. Moreover, existing studies lack a comprehensive exploration of how the composition of initiator and the chemistry of resin influence the stability of FP propagation, especially in scenarios requiring minimal energy input, such as space manufacturing. To address these gaps, this study focuses on a novel RICFP system combining DGEBA epoxy resin with dual initiators, leveraging the synergistic effects of radical-induced acid generation and cationic propagation. This approach aims to enhance the controllability of reaction, while maintaining the self-sustaining characteristics that are critical for in-orbit applications.

This work established a curing kinetics model for a kind of epoxy resin RICFP system and investigated key parameters influencing the frontal polymerization reaction, including the pre-exponential factor and activation energy. The polymerization rate was controlled by adjusting the ratio of initiator content to alicyclic resin content. Furthermore, finite element analysis was employed to simulate the curing and heat transfer processes associated with frontal polymerization, evaluating the sensitivity of the polymerization rate to various process parameters. This research provides a numerical foundation for the engineering fabrication of large-scale components in complex space environments.

The primary objectives of this work are as follows: (1) to establish a curing kinetics model for the RICFP epoxy system, elucidating the relationship among activation energy, pre-exponential factor and reaction rate, (2) to investigate how initiator concentrations and alicyclic resin influence polymerization speed and the properties of cured resin, and (3) to develop and validate a finite element simulation framework for predicting FP behavior under different processing and environmental conditions. We hypothesize that optimizing the content of the initiator can facilitate precise control over polymerization rate and reduce temperature gradients. Furthermore, it is proposed that incorporating alicyclic resins will enhance the stability of reaction propagation and the performance of cured resin. These advancements aim to provide a foundational framework for scalable, energy-efficient FP processes in space manufacturing.

## 2. Experiment and Methods

### 2.1. Materials

**Figure 1:**
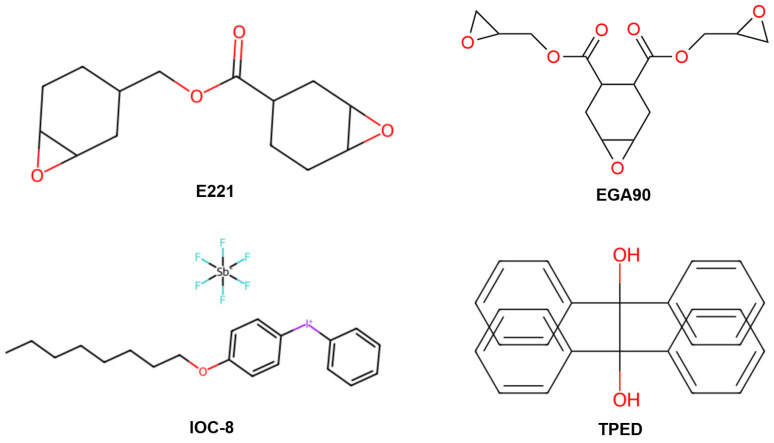
Chemical formulas of various materials used in this work.

The chemical reagents used in this study are shown in Figure 1. DGEBA E51 epoxy resin was produced by Nantong Xingchen Synthetic Material Co., Ltd. (Nantong, China) 3,4-epoxycyclohexylmethyl-3′,4′-epoxycyclohexane carboxylate (E221) and 4,5-epoxyhexane-1,2-dicarboxylic acid diglycidyl ester (EGA90) were supplied by Aladdin Chemical Reagent Corporation (Shanghai, China). p-(octyloxyphenyl) phenyl iodonium hexafluoroantimonate (IOC-8) and 1,1,2,2-tetraphenyl ethanediol (TPED) were used as acid photogenerator (PGA) and thermal radical initiator (RTI), respectively.

### 2.2. Experiment and Simulation

#### 2.2.1. RICFP Set-Up

The preparation steps for frontal polymerization epoxy resin are as follows: First, weigh the required amount of epoxy resin and place it in a beaker. According to the specifications of the reaction system, add a suitable amount of photo initiator and thermal initiator. Subsequently, place the beaker in a 50 °C oil bath and initiate stirring to ensure the uniform dissolution and thorough dispersion of the initiators within the resin matrix. A temperature of 50 °C was selected for the resin-initiator mixture to ensure complete dissolution of the thermal initiator (TPED) and the photoacid generator (IOC-8) and avoid premature thermal decomposition of the RTI, which occurs above 60 °C. This balance ensures homogeneity and stability of the uncured resin, enabling reliable initiation under UV exposure. The stirring should continue for approximately 120 min to ensure complete dissolution of the initiators and the formation of a homogeneous mixture. Afterward, place the container with the frontal polymerization resin in a vacuum oven and evacuate for 15 min to remove any bubbles generated during the stirring process and present within the resin itself.

**Figure 2:**
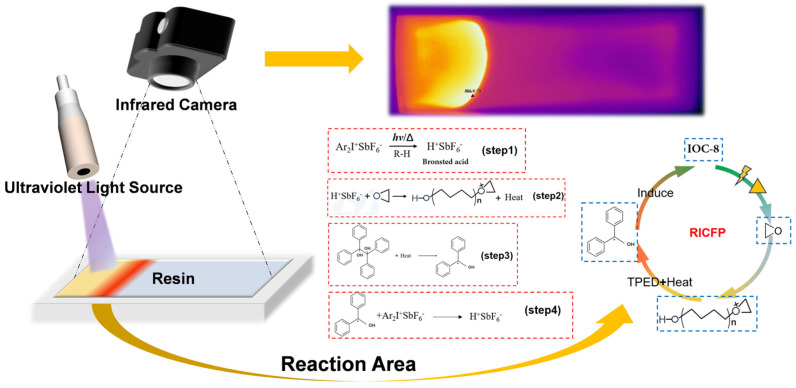
Radical-induced cationic frontal polymerization (RICFP) curing procedure and testing scheme.

Figure 2 illustrates the schematic diagram of the measurement method for the propagation rate of frontal polymerization. First, the prepared frontal polymerization epoxy resin was transferred into a custom-made silicone rubber mold with dimensions of 100 mm × 40 mm × 12 mm (length × width × height). The custom silicone mold dimensions were chosen to mimic small-scale structural components, while the infrared thermal imaging provided spatially resolved temperature data to validate front propagation kinetics. A UV light source was continuously applied at one end of the resin until the resin at that location began to cure. The UV light source was then promptly removed, and the infrared thermal imaging camera was used to monitor the progression of the polymerization front.

The propagation rate of the frontal polymerization can be calculated using Formula (1):

(1) $$V_{f}=\frac{x}{t}$$

where *V_f_* represents the propagation rate of the polymerization front, *x* is the distance moved by the polymerization front, and *t* is the elapsed time for the polymerization. The polymerization rate is typically expressed in units of cm·min^−1^.

Additionally, the maximum temperature of the polymerization front was obtained from the data recorded by the infrared thermal imaging camera, which was also used to evaluate the frontal polymerization.

#### 2.2.2. Differential Scanning Calorimetry (DSC)

DSC was employed to quantify the curing kinetics due to its ability to measure heat flow during exothermic reactions with high sensitivity. This method allows for the precise determination of activation energy (*E_a_*) and pre-exponential factor (*A*) through non-isothermal analysis at multiple heating rates, which is critical for modeling self-sustaining reactions in FP systems. The nitrogen atmosphere prevented oxidative degradation, ensuring data accuracy.

The start and end reaction temperatures, as well as the exothermic heat release during the curing reaction of the frontal polymerization epoxy resin, were characterized using DSC with a NETZSCH DSC 214 POLYMA (NETZSCH, Selb, Germany) under nitrogen atmosphere, ranging from ambient temperature to 250 °C. The heating rates used in the experiments included 5, 10, 15, 20 and 25 °C·min^−1^.

#### 2.2.3. Dynamic Mechanical Analysis (DMA)

DMA was utilized to assess viscoelastic properties of cured resins, as it directly correlates with structural performance in space environments. It can provide insights into glass transition temperature (*T_g_*) and crosslink density, which are critical for evaluating thermal stability under operational stresses.

Dynamic mechanical properties of the cured frontal polymerization epoxy resin were tested using a NETZSCH DMA 242 E dynamic thermomechanical analyzer (NETZSCH, Selb, Germany). The cured resin samples were sized at 40 mm × 6 mm × 2 mm (length × width × height) and were tested under nitrogen atmosphere at a heating rate of 5 °C·min^−1^. The temperature range was from room temperature to 250 °C, and the testing mode was three-point bending.

#### 2.2.4. Fourier Transform Infrared Spectroscopy (FTIR) Testing

FTIR spectroscopy was applied to monitor chemical changes during curing, particularly the disappearance of epoxy rings (914 cm^−1^) and formation of ester linkages (1730 cm^−1^). This technique was chosen for its molecular specificity, enabling real-time tracking of reaction progress and validation of the RICFP mechanism. The testing wavenumber range was 400–4000 cm^−1^.

#### 2.2.5. Finite Element Simulation Method for Heat Transfer During Curing Process of Frontal Polymerization

**Figure 3:**
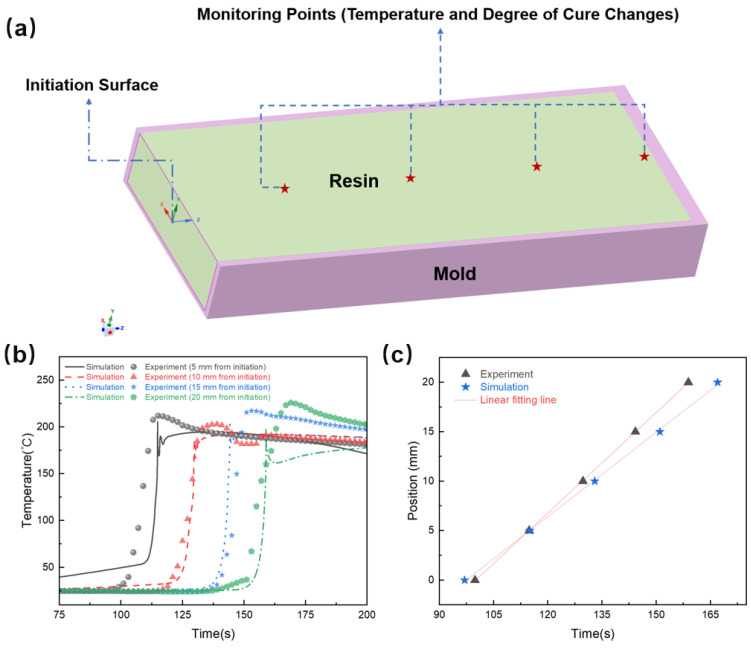
(**a**) Geometric model for monitoring heat transfer during frontal polymerization. The red stars indicate the monitoring points for temperature and degree of cure. Comparison between simulation and experiment of the frontal polymerization process: (**b**) temperature variation over time at different positions and (**c**) position of front movement over time.

Finite element analysis (FEA) was selected to simulate heat transfer and curing dynamics, because it accommodates complex geometries, anisotropic material properties and transient boundary conditions, which need to be considered in space manufacturing. The model integrates Fourier’s law with an internal heat source term (Equation (2)) to capture the coupling between exothermic curing and thermal diffusion. ANSYS Fluent 2024 R1 was chosen for its robust solver capabilities in handling reaction–diffusion systems. Validation against experimental temperature profiles (Figure 3b,c) confirms the reliability of the model for predicting polymerization front behavior under varying process parameters.

The finite element model integrates Fourier’s law of heat conduction (Equation (2)) with an internal heat source term derived from the curing kinetics (Equation (6)). This approach is consistent with methodologies validated in previous studies on FP systems [23], ensuring alignment with these established computational frameworks. The governing equations were solved using ANSYS Fluent, leveraging its robust transient solver and user-defined function (UDF) capabilities to couple reaction kinetics with heat transfer.

(2) $$\frac{\partial (\rho_{r}C_{r}T)}{\partial t}=\frac{\partial }{\partial x}\left(k_{x}\frac{\partial T}{\partial x}\right)+\frac{\partial }{\partial y}\left(k_{y}\frac{\partial T}{\partial y}\right)+\frac{\partial }{\partial z}\left(k_{z}\frac{\partial T}{\partial z}\right)+\rho_{r}\dot{H}$$

In the equation, *ρ_r_* is resin density, *C_r_* is specific heat capacity of the resin, *T* is absolute temperature, *t* is curing time and *k_x_*, *k_y_*, *k_z_* are thermal conductivities of the resin in the material coordinate system, as shown in Figure 3a. $\dot{H}\;$ represents heat release rate from the reaction, which is related to the curing reaction rate and satisfies the following equation:

(3) $$\dot{H}=H_{R}\frac{d\alpha }{dt}$$

where *H_R_* is total curing enthalpy change of the resin, and *α* is degree of curing. The curing rate can be obtained from curing kinetics parameters of the resin.

The established geometry model is shown in Figure 3a. The resin and mold constitute the primary structure of the model, with the left side designated as the initiation surface, serving as the starting point for the polymerization reaction. The reaction initiates at this surface and propagates through the resin via a self-sustaining heat transfer reaction process. To facilitate real-time observation of the propagation characteristics of the reaction front, multiple monitoring points are set within the model to record the changes in temperature and degree of cure. In the simulation, the resin cross-section dimensions were set to 40 mm × 12 mm (width × height), with the mold wall thickness set to 2 mm. The initial temperature of the resin and the ambient temperature were both set to room temperature (25 °C). The initiation of the reaction was controlled by regulating the temperature of the initiation surface, with the temperature parameters derived from the measured values in Section 2.2.1. Table 1 presents the thermal physical parameters of the epoxy resin and two kinds of mold materials used in the finite element simulation.

To verify the reliability of the finite element simulation results, experimental validation was conducted under identical conditions. During the experiment, thermocouples and a temperature inspection device were used to track the temperature changes at four locations. The comparison between the experimental and simulation results is shown in Figure 3b,c. In the figures, the solid lines represent the simulated temperature change curves, and the scatter points correspond to the experimental data. The simulation and experimental results demonstrate a high degree of consistency during the rapid temperature rise phase, indicating that the finite element model accurately captures the heat-release and temperature-rise behavior associated with the polymerization reaction. As the monitoring point location increases from 5 mm to 20 mm, the time taken for the temperature to rise progressively extends. From Figure 3c, it can be seen that the position of the front shifts linearly over time. It indicates that the time intervals for the temperature rising between adjacent monitoring points remain approximately constant. This consistent behavior, evident in both the simulation and the experimental data, reflects the characteristic of the polymerization front advancing at a relatively stable rate over greater distances.

The validation of the heat transfer model against experimental data underscores its applicability for predicting polymerization behavior. However, the model simplifies the curing kinetics of resin to a single-step Arrhenius equation, which is not suitable for multi-stage reaction mechanisms.

## 3. Results and Discussion

### 3.1. Curing Mechanism of Frontal Polymerization

The radical-induced cationic frontal polymerization (RICFP) mechanism used in this study is shown in Figure 1. The reaction is initiated by ultraviolet (UV) light exposure. First, diaryl iodonium salt (Ar_2_I^+^SbF_6_^−^, IOC-8) as PAG decomposes under light (*hν*) or heat (Δ) conditions to generate strong Brønsted acid, H^+^SbF_6_^−^. The strong acid then reacts with epoxy groups in the monomer, causing the epoxy groups to open and form intermediates with cationic active centers. This ring-opening reaction releases energy and generates new active centers, thereby initiating the cationic polymerization process. Additionally, the TPED molecules at the initiation site act as RTI and decompose under the heat released from the ring-opening reaction to generate free radicals. These free radicals continue to react with IOC-8, leading to more generation of H^+^SbF_6_^−^, which subsequently reacts with residual epoxy groups, thereby sustaining the polymerization front. This step establishes a coupling mechanism between free radical and cationic polymerization, imparting a robust self-sustaining characteristic to the reaction. Consequently, the polymerization front continues to propagate through free radical generation, cationic activation and chain growth. The heat released during the reaction, combined with the cycling of free radicals and superacid, ensures stable progression of the reaction without the requirement for continuous external energy input. This self-sustaining nature significantly reduces the need for additional heat sources, achieving efficient progression of the polymerization front and rapid curing.

### 3.2. Curing Kinetics of Frontal Polymerization

**Figure 4:**
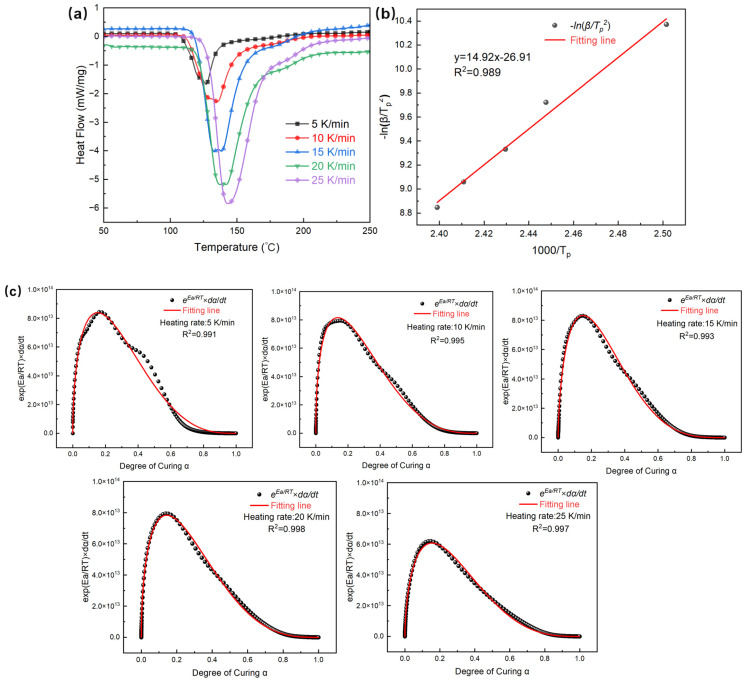
(**a**) DSC curves for E51 epoxy resin with 1 wt.% of both PAG and RTI at different heating rates. (**b**) Curve of −ln(*β*/*T_p_*^2^) versus 1000/*T_p_* for the E51 resin system. (**c**) Fitting curves based on the Kamal reaction model of the E51 resin system under different heating rates.

The propagation of the RICFP polymerization front relies on the accumulation and transfer of reaction heat, resulting in a significant temperature dependence of both the curing rate and the front propagation rate. Figure 4a shows the DSC curves for epoxy resin E51 resin with 1 wt.% of both PAG and RTI at different heating rates. The heat-release peaks are observed within the temperature range of 100 °C to 220 °C. As the heating rate increases, these peaks shift towards higher temperatures and exhibit sharper characteristics.

The curing kinetics model for thermosetting resins is typically based on the Kissinger approximation to calculate apparent activation energy of curing reaction, which reflects initial stages of the non-isothermal curing process.

A comparative analysis of kinetic models (Kissinger vs. Ozawa/Friedman) highlights the suitability of the Kissinger method for non-isothermal RICFP systems. Unlike the isoconversional approach of the Ozawa method and the differential method of the Friedman model, the Kissinger model focuses on the peak temperature on the DSC (*T_p_*), so it ensures robustness to experimental noise and simplifies parameter extraction. The derived *E_a_* and *A* values are integral to the Kamal autocatalytic model, which accurately predicts FP propagation rates. This approach obtains a balance between methodological rigor and computational efficiency, aligning with the emphasis of the study on rapid, self-sustaining curing mechanisms.

The linearized form of the Kissinger model is

(4) $$\ln\left(\frac{\beta }{{T_{p}}^{2}}\right)=ln\left(\frac{Q_{p}AR}{E_{a}}\right)-\frac{E_{a}}{RT_{p}}$$

In the equation, *β* is the heating rate, *T_p_* is the peak temperature on the DSC curve of the resin, *A* is the pre-exponential factor, *E_a_* is the apparent activation energy and *R* is the universal gas constant, which is 8.314 J·mol^−1^·K^−1^. By plotting −ln(*β*/*T_p_^2^*) as the vertical axis and 1000/*T_p_* as the horizontal axis, a linear fit was conducted to obtain the slope *E_a_*/*R*, thereby calculating *E_a_*. The linear regression in Figure 4b yields an *R*^2^ value of 0.989, indicating a strong linear relationship between ln(*β*/*T_p_*^2^) and 1/*T_p_*. The predictive capability of the model was further validated through its integration with the Kamal autocatalytic kinetics framework, which accurately simulated polymerization front propagation. The slope leads to an *E_a_* of 124.08 kJ·mol^−1^ for the frontal polymerization system.

Frontal polymerization is generally more effectively modeled using a self-catalyzed model due to the mechanism of self-sustaining heat generation during the process. This mechanism frequently induces a localized increase in temperature at the reaction front, thereby accelerating the polymerization. Consequently, this self-sustaining feature often results in a self-catalytic effect, wherein the heat produced by the reaction further promotes the polymerization of unreacted monomers. Therefore, the self-catalytic model provides a more accurate description of the characteristic acceleration of the polymerization rate in relation to the increasing degree of cure within the frontal polymerization system.

By incorporating the autocatalytic model into the relationship between the curing reaction rate and activation energy, the curing kinetics equation of the autocatalytic model can be obtained, expressed as

(5) $$exp(\frac{E_{a}}{RT})\frac{d\alpha }{dt}=A{\alpha^{m}(1-\alpha )}^{n}$$

In this equation, *m* and *n* represent the reaction orders. The left part of Equation (5) can be calculated using the DSC heating curve and the obtained apparent activation energy. The right part of the equation consists of unknown parameters in the curing kinetics model, including the pre-exponential factor *A*, and the reaction orders *m* and *n*.

Therefore, after calculating the relevant parameters, the curve of the left part of the equation with respect to the degree of curing can be constructed. Then, the fitting is performed using the equation of the right part (the fitting results are shown in Figure 4c), which allows for the determination of the unknown curing kinetics model parameters *A*, *m* and *n*. The data are shown in Table 2.

Finally, the arithmetic average of the parameters fitted at the five heating rates is taken as the final determined model parameters. Combined with the obtained activation energy parameters, the curing kinetic equation for the E51 epoxy resin is derived as follows:

(6) $$\frac{d\alpha }{dt}=3.59311\times 10^{14}e^{\left(\frac{{-124.07\times 10}^{3}}{RT}\right)}{\alpha^{0.53832}(1-\alpha )}^{3.10371}$$

**Figure 5:**
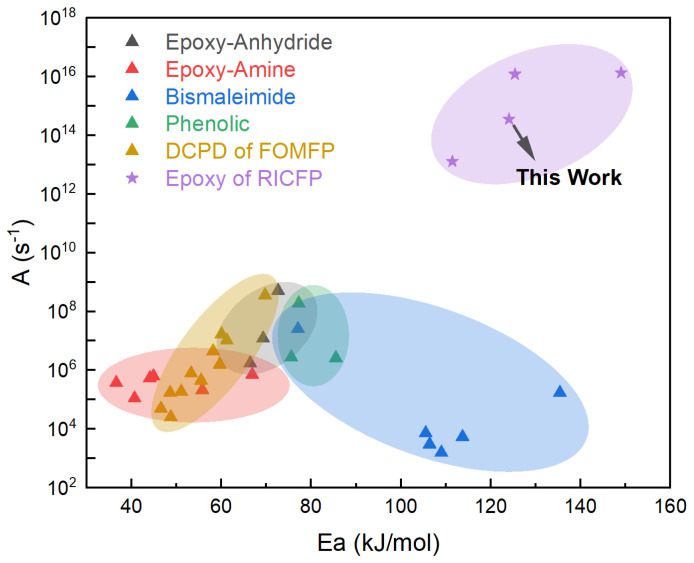
Relationship between activation energy and polymerization rate pre-exponential factor for different resin reaction systems [24,25,26,27,28,29,30,31,32].

Figure 5 shows the relationship between the activation energy and the pre-exponential factor of the polymerization rate for different resin systems. From the figure, it can be observed that the activation energy range for the studied RICFP is relatively high (approximately 100–140 kJ·mol^−1^), indicating that the reaction requires higher energy input to initiate, which results in an elevated reaction threshold. However, once the reaction is activated, the pre-exponential factor (*A*) for the RICFP (ranging from 10^12^ to 10^16^ s^−1^) is significantly higher than those of other systems, enabling a faster polymerization rate and accelerating the propagation of the polymerization front. This combination of high activation energy and high reaction rate renders RICFP particularly suitable for applications requiring rapid curing and low-temperature stability. Therefore, RICFP exhibits distinct characteristics of high reaction threshold and high reaction rate.

### 3.3. Influence of Resin Composition on Frontal Polymerization Behavior

#### 3.3.1. Effect of Initiator Concentration on Polymerization Rate

##### Effect of PAG Concentration on Polymerization Rate

**Figure 6:**
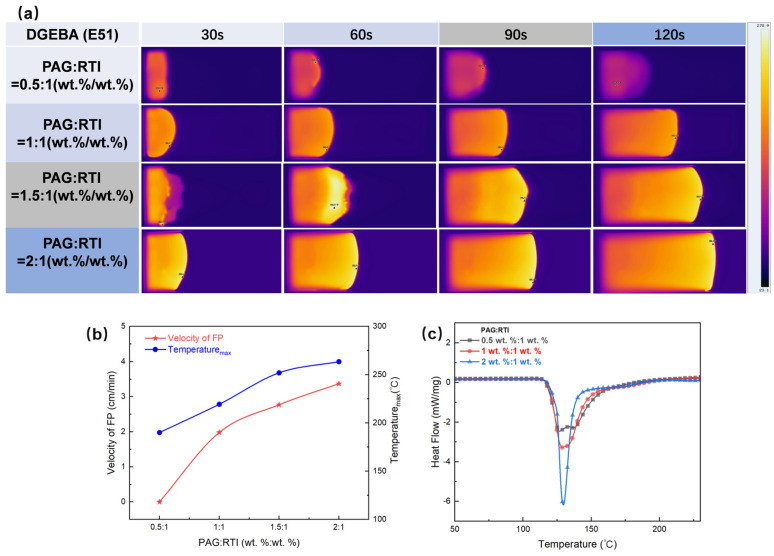
(**a**) Effect of PAG concentration on the frontal polymerization rate. (**b**) Polymerization rate and maximum temperature curves for different PAG concentrations during frontal polymerization. (**c**) DSC exothermic curves for different PAG contents.

Figure 6a presents infrared thermal imaging of the E51 resin RICFP system at various time points, incorporating different concentrations of PAG while maintaining a constant concentration of RTI. It can be seen that, under the condition of PAG:RTI = 0.5 wt.%:1 wt.%, polymerization only occurs near the initiation surface at both 30 s and 60 s, without the establishment of a complete polymerization front. Additionally, at 90 s and 120 s, the polymerization phenomenon ceases, indicating the termination of the polymerization reaction. This observation suggests that the concentration of the initiators is insufficient to sustain the continuous progression of the reaction.

When the PAG:RTI concentration is 1 wt.%:1 wt.%, the polymerization reaction is significantly enhanced, forming a complete polymerization front from 60 s to 120 s. As the concentration increases to 1.5 wt.%:1 wt.% and 2 wt.%:1 wt.%, both the propagation speeds of the polymerization front and the reaction temperatures increase significantly. Thus, it can be concluded that the PAG concentration has significant impacts on the sustainability, propagation speed and temperature distribution of the polymerization front. At lower concentration, sustaining the polymerization is difficult, while higher concentration greatly enhances the advancement of the polymerization reaction. As seen in Figure 6b, with the increase in PAG content, the peak temperature of the polymerization front rises accordingly. This indicates that higher concentrations of the initiator increase reaction activity, resulting in elevated system temperatures.

To further investigate the specific mechanism of the role of PAG in the polymerization process, DSC exothermic behavior was evaluated at different PAG:RTI concentrations (0.5 wt.%:1 wt.%, 1 wt.%:1 wt.%, and 2 wt.%:1 wt.%), as shown in Figure 6c. With the increase in PAG concentration, the intensity of the exothermic peak significantly increases, indicating that higher concentrations of initiator effectively trigger the polymerization reaction and yield a more intense reaction. Meanwhile, under low PAG concentration (0.5 wt.%), the exothermic peak is relatively flat, suggesting a lower reaction rate. In contrast, at higher PAG concentrations (1 wt.% and 2 wt.%), the exothermic peaks in the DSC curves become sharper, reflecting that the polymerization reaction occurs more intensively with a higher reaction rate. Therefore, by adjusting the PAG concentration, the polymerization rate and the heat-release behavior of the epoxy resin can be effectively controlled.

##### Effect of RTI Concentration on Polymerization Rate

**Figure 7:**
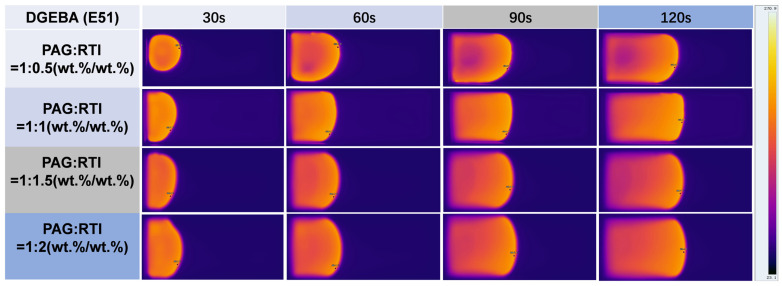
Effect of thermal initiator concentration on the frontal polymerization rate.

The main function of RTI is to generate free radical activators through thermal decomposition during the RICFP process, which in turn induce the PAG to initiate the ring-opening reaction of the epoxy resin and release heat. Figure 7 shows infrared thermal imaging of the E51 resin RICFP system with different RTI concentrations at a fixed PAG concentration. From the thermal imaging and the data listed in Table 3, it can be observed that the variation in RTI concentration has a minimal effect on the polymerization rate and peak temperature. Regardless of the RTI concentration, the expansion of the high-temperature region and the propagation speed of the polymerization front from 30 s to 120 s are almost identical. In addition, the polymerization reaction at high RTI concentrations (e.g., 1 wt.%:1.5 wt.% and 1 wt.%:2 wt.%) does not show a significant acceleration effect compared with that at low RTI concentrations (e.g., 1 wt.%:0.5 wt.%).

Table 3 and thermal imaging data (Figure 7) reveal that the variations in RTI concentration (0.5–2 wt.%) have a minimal impact on both polymerization rate (*V_f_*) and peak temperature (*T*_max_). It is attributed to radical saturation beyond 0.5 wt.% RTI, where acid generation becomes rate limited by IOC-8 decomposition.

#### 3.3.2. Effect of Alicyclic Resin on Polymerization Rate

**Figure 8:**
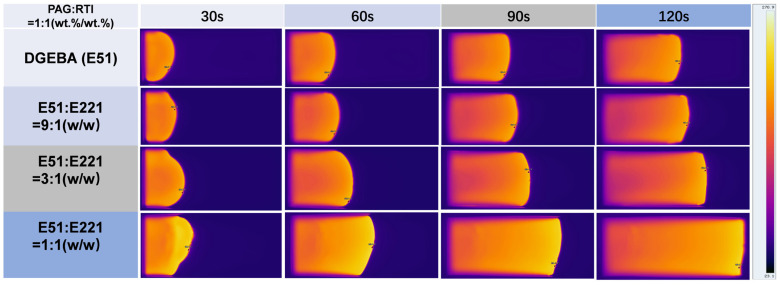
Effect of difunctional alicyclic epoxy resin E221 content on the frontal polymerization rate.

In this study, two kinds of alicyclic resins, E221 and EGA90, were utilized to modify the properties of the E51 resin RICFP system. Figure 8 shows infrared thermography images of various resin systems with different difunctional alicyclic E221 resin content at various time points. From the figure, it can be observed that as the E221 content increases, the polymerization speed significantly accelerates and the reaction area progressively shifts to the right within the same time frame. It indicates that E221 obviously promotes the reaction and accelerates the advancement of the frontal polymerization.

**Figure 9:**
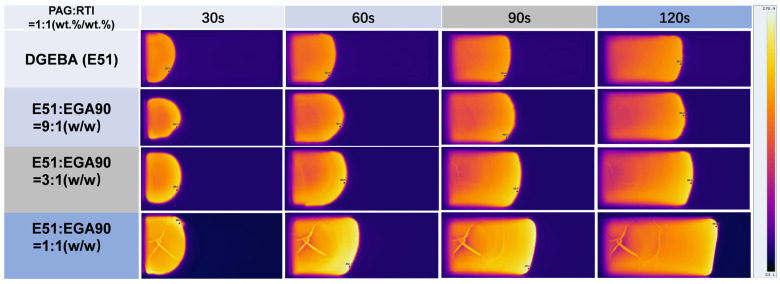
Effect of multifunctional alicyclic epoxy resin EGA90 content on the polymerization rate.

Furthermore, the effect of multifunctional alicyclic EGA90 resin on the frontal polymerization was studied, and the temperature distributions for different EGA90 content are shown in Figure 9. It demonstrates that higher EGA90 content leads to higher frontal polymerization rate. In the infrared thermography images at different time points, a ratio of EGA90 to 1:1 (*w*/*w*) that approaches unity indicates a more rapid expansion of the polymerization front. In comparison to the case involving the addition of E221, the enhancing effect of EGA90 on the polymerization rate is less significant.

Additionally, a comparison of the polymerization rates and peak temperatures of the two cycloaliphatic resins reveals that, for the same cycloaliphatic resin content, the increase in polymerization rate with EGA90 is less pronounced than that with E221 resin. However, the peak temperature of the EGA90 resin system is higher than that of the E221 resin system at the same resin content.

**Figure 10:**
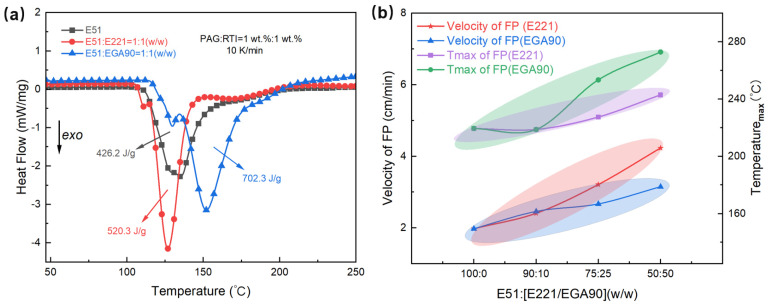
(**a**) DSC exothermic curves and (**b**) polymerization rate vs. peak temperature curves for different cycloaliphatic epoxy resins.

Based on the data in Figure 10, it can be seen that E221 and EGA90 resins exhibit different behaviors of polymerization. From the DSC exothermic curves in Figure 10a, it is evident that the exothermic heat of the system significantly increases after the addition of cycloaliphatic resins. The exothermic heat of pure E51 resin is 426.2 J·g^−1^, while the exothermic heat increases to 520.3 J·g^−1^ and 702.3 J·g^−1^ after adding E221 and EGA90, respectively. However, these two resins show differences in the peak temperatures of the exothermic curves. The DSC peak temperature of the E221 system is inclined towards the lower temperature range, while the peak temperature of the EGA90 system shifts towards the higher temperature range. Therefore, the introduction of E221 not only increases the exothermic heat, but also results in an earlier appearance of the exothermic peak. These two factors work together to significantly accelerate the polymerization rate. In contrast, the addition of EGA90 shifts the exothermic peak to higher temperature, but its increased exothermic heat still contributes to an increasing polymerization rate.

Figure 10b distinctly illustrates the different impacts of the two resins. As the ratios of E221 and EGA90 change, the slopes of the changes in the polymerization rate and peak temperature vary, reflecting the degree of contribution of the resin composition to each parameter. A larger slope in polymerization rate for E221 indicates a more significant effect on the polymerization rate, while a larger slope in peak temperature for EGA90 suggests that it has a greater contribution to the peak temperature.

This divergence arises from fundamental differences in their molecular architectures. The alicyclic structure of E221 imposes significant ring strain, lowering the activation energy for cationic ring-opening polymerization. The rigid cyclohexane backbone facilitates rapid protonation by the superacid (generated from PAG decomposition), enabling faster propagation of the curing front. Additionally, the absence of bulky substituents minimizes steric hindrance during chain growth, as reported in studies of alicyclic epoxies by Zhou et al. [19]. In contrast, linear ester groups of EGA90 introduce electron-withdrawing effects, reducing the electrophilicity of the epoxy groups and slowing protonation kinetics. The flexible aliphatic chain further dissipates thermal energy, delaying the formation of a stable reaction front. Moreover, the ester moieties create steric barriers that impede access to the epoxy rings.

### 3.4. Effect of Different Polymerization System on Dynamic Mechanical Property of FP Resin

**Figure 11:**
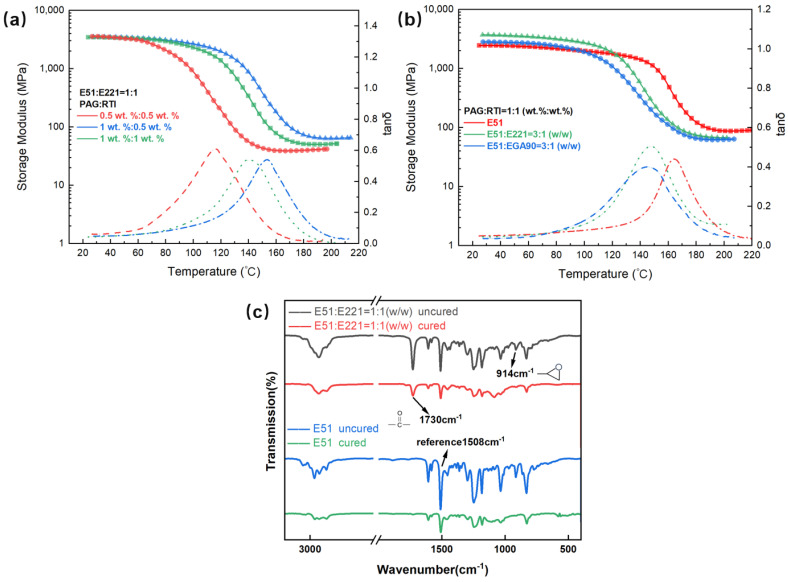
(**a**) Effect of initiator concentration on DMA properties of the cured resin. (**b**) Effect of alicyclic resin on DMA properties of the cured resin. The solid lines represent the variation of the storage modulus of the cured material with temperature, while the dashed lines represent the variation of the loss factor (tan α) with temperature. (**c**) FTIR spectra of E51 with E221 system before and after UV-induced frontal polymerization.

Figure 11a shows the DMA curves of the cured resin with E51:E221 = 1:1 (*w*/*w*) at different initiator concentrations. It can be observed that the initial storage modulus does not vary significantly with different initiator contents, remaining consistently above 3400 MPa. However, the *T_g_* decreases significantly as the PAG concentration decreases. In contrast, the variation in RTI concentration has a smaller effect on *T_g_*.

Figure 11b shows the DMA curves of the cured resins with different cyclic aliphatic resin contents. It demonstrates that cyclic aliphatic resins have significant impacts on the initial storage modulus and glass transition temperature. In terms of the initial storage modulus, the E51:E221 = 3:1 (*w*/*w*) system exhibits a higher storage modulus of 3713 MPa compared to pure E51 resin, which has a modulus of 2462 MPa. For the E51:EGA90 system with a weight ratio of 3:1, the initial storage modulus exhibits a slight decline to 2826 MPa. Nevertheless, this value remains higher than that of the pure E51 system. The phenomenon can be analyzed as follows [33,34]: Cyclic aliphatic epoxy resins, such as E221 and EGA90, typically exhibit a more rigid structure due to their cyclic molecular backbone. This rigidity results in a higher initial storage modulus. Furthermore, increased ring strain and epoxy value contribute to greater reactivity, which significantly enhances crosslink density during the curing process, thereby increasing the storage modulus.

The *T_g_* of the pure E51 system is 164.1 °C, whereas in the E51:E221 = 3:1 (*w*/*w*) system, *T_g_* decreases to 147.8 °C. In the E51:EGA90 = 3:1 (*w*/*w*) system, it decreases to 145.0 °C. It indicates that the addition of cyclic aliphatic resins leads to a decrease in the thermal stability of the FP resin. The observed decrease in *T_g_* can be attributed to the differences in reactivity between bisphenol A-type epoxy resin and cyclic aliphatic resin. It results in the formation of a heterogeneous crosslinking network, which disrupts the close packing of polymer chains and thereby increases the free volume. This structural characteristic enhances molecular segment mobility at lower temperatures, ultimately leading to a reduction in *T_g_*. To quantify and analyze this effect, the infrared spectra of the E51 and E51:E221 = 1:1 (*w*/*w*) systems before and after curing were compared. The FTIR spectra (Figure 11c) reveal chemical distinctions between the E221 and E51 systems. For E221, the 1730 cm^−1^ peak corresponds to carbonyl group (C=O) of its cyclohexenyl carboxylate ester moiety, with no intensity change before and after curing, confirming non-involvement of the carbonyl group in reactions. E51 lacks the 1730 cm^−1^ peak due to the absence of carbonyl structures. In contrast, significant attenuation of the 914 cm^−1^ peak (epoxy C-O-C) confirms cationic ring-opening-dominated curing mechanisms. As shown in Figure 11c, there is a significant change in the intensity of the absorption peak at 914 cm^−1^ for the epoxy groups before and after curing. This change reflects the influence of cyclic aliphatic resins on the conversion rate of the epoxy groups. The results indicate that the incorporation of cyclic aliphatic resins substantially decreases the epoxy group conversion rate, decreasing it from 91.6% to 72.9%, suggesting that a considerable proportion of epoxy groups remain unreacted. This outcome further verifies the formation of a heterogeneous crosslinking network, which may weaken the thermal stability of the material and contribute to the reduction in *T_g_*.

### 3.5. Simulation of the Curing and Heat Transfer Process in Frontal Polymerization

#### 3.5.1. Effect of Molding Factors on Polymerization Rate

**Figure 12:**
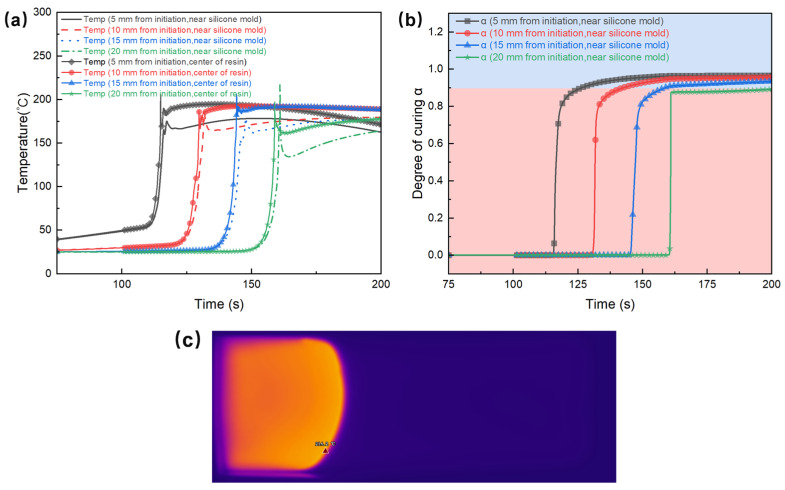
(**a**) Simulated temperature variation over time at the center of the E51 resin system and near the silicone rubber mold. (**b**) Simulated degree of cure variation over time at different positions. The red background indicates a degree of cure below 90%, while the blue background represents a degree of cure above 90%. (**c**) Infrared thermography of the frontal polymerization process.

In order to thoroughly investigate the curing and heat transfer process of the studied FP E51 resin system with PAG:RTI = 1:1 (*w*/*w*), a numerical simulation was conducted by means of the finite element method. Figure 12a shows the temperature differences between the center of the resin and the area near the silicone rubber mold during the frontal polymerization process. The silicone rubber mold exhibits higher thermal conductivity than the resin, resulting in more pronounced temperature fluctuations near the mold, characterized by a lower peak temperature and a more rapid cooling rate. The final data point, located near the mold, shows a higher peak temperature, possibly attributable to the boundary effect. This disparity in temperature distribution significantly influences both the polymerization rate and the ultimate curing state of the frontal polymerization reaction.

In addition, Figure 12c shows the infrared thermal imaging of the frontal polymerization process in a silicone rubber mold for the E51 resin system. In conjunction with the simulation results presented in Figure 12a, it is evident that the polymerization front near the sides of the mold exhibits a notable delay when compared to the center of the resin. The phenomenon results in an arc-shaped advancement of the polymerization front, as observed in the infrared thermal imaging. Figure 12b shows the curing degree curves over time at the points adjacent to the mold in the simulation. It can be seen that the curing degree at all four locations has not reached 100%.

**Figure 13:**
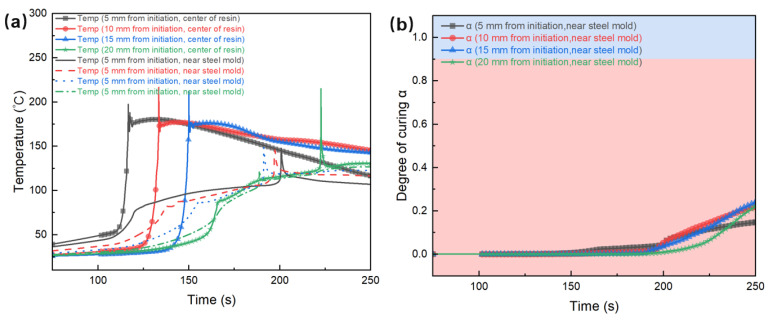
(**a**) Simulated temperature variation over time during frontal polymerization at the center of the E51 resin system and near a steel mold. (**b**) Simulated degree of cure curves using the steel mold. The red background indicates a degree of cure below 90%, while the blue background represents a degree of cure above 90%.

The simulation method was used to investigate the influence of a steel mold on the polymerization behavior, as shown in Figure 13. It can be observed that in regions adjacent to the steel mold, the rate of temperature increase is slower, and the peak temperature is significantly lower than that at the center, which is below 150 °C. This phenomenon can be attributed to the high thermal conductivity of the steel mold, which facilitates rapid heat dissipation and consequently suppresses the polymerization reaction in these regions. Figure 13b illustrates the variation in the curing degree near the steel mold, revealing that the cure degrees at all four monitored points remain below 30% by the end of the reaction. It indicates that the heat dissipation effect of the steel mold significantly impacts the temperature distribution and reaction rate, with its influence being more pronounced than that of the silicone rubber mold.

#### 3.5.2. Effect of Resin Cross-Sectional Dimensions on Polymerization Rate

**Figure 14:**
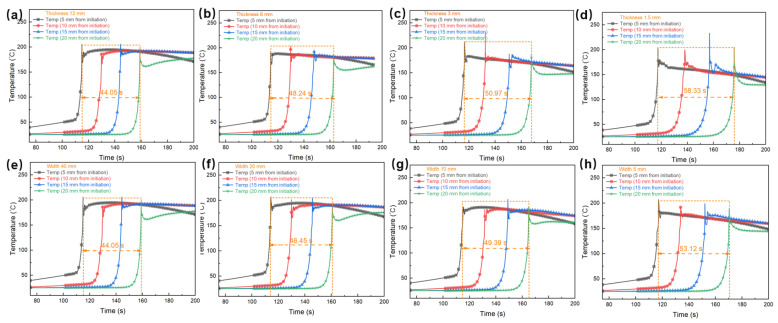
(**a**–**d**) Temperature and degree of cure variation over time for the E51 resin system with different section thicknesses and (**e**–**h**) different section widths. The orange dashed lines represent the time intervals between the first monitoring point and the fourth monitoring point.

Considering the significance of heat generated during the curing process of FP resin, which contributes to its self-sustaining curing property, it is essential to take the dimensions of the FP resin into account. The cross-sectional area of the resin is determined by both its thickness and width. Figure 14 illustrates the simulation results of frontal polymerization conducted under varying conditions of thickness and width, with each parameter controlled in separate cases. All simulations were performed utilizing a silicone rubber mold.

In addition, as the thickness decreases, some monitoring points exhibit unstable peak temperature behavior, with instances of “overheating”, as observed at the peak temperatures at a distance of 10 mm from the initiation in Figure 14c and at a distance of 15 mm from the initiation in Figure 14d. The reduction in polymerization rate associated with decreased thickness is primarily attributed to the lower overall heat capacity of the material, which reduces the accumulation of heat released during the polymerization reaction within the resin. Consequently, in thinner resin layers, the heat available to sustain the polymerization front is limited, resulting in a slower polymerization rate. Additionally, thinner resin cross-sections have difficulty effectively dissipating the heat generated by the reaction, causing rapid heat accumulation in highly reactive local regions, which leads to a sudden spike in temperature. This localized heat accumulation further intensified by the shorter heat dissipation path in thin-layer materials, while the reduced thermal dissipation efficiency amplifies localized temperature instability. Moreover, thinner resin layers are more dependent on surface heat dissipation to the mold or surrounding air, making them more susceptible to temperature fluctuations resulting from uneven local heat dissipation, which can lead to overheating phenomena. Therefore, the reduction in resin thickness, which leads to a decrease in polymerization rate and localized overheating phenomena, highlights thermal imbalance in thin-layer polymerization systems.

Similarly, as shown in Figure 14e–h, the time required for the polymerization front propagation gradually increases, and the polymerization rate decreases with a reduction in resin cross-sectional width. Specifically, at a cross-sectional width of 40 mm, the propagation time interval of the polymerization front is 44.1 s. However, when the width is decreased to 5 mm, this interval extends to 53.1 s. Similar to the observed trend related to thickness variation, a decrease in width leads to a reduction in the polymerization rate. Notably, unlike the scenario involving thickness variation, the temperature curves do not exhibit significant “overheating” phenomena.

In conjunction with the geometric model constructed in Figure 3, both sides in the width direction are in contact with molds, facilitating heat dissipation through the highly thermally conductive mold materials, thereby controlling the temperature rise in the resin. However, in the thickness direction, only the bottom is in contact with a mold, while the top is exposed to air, where heat dissipation occurs primarily through weaker convection. As a result, the heat dissipation at the bottom is more efficient compared to the top, making the exposed resin layer on the top more prone to heat accumulation.

This difference in heat transfer conditions implies that the molds in the width direction help to prevent lateral “overheating” phenomena, while in the thickness direction, heat dissipation relies exclusively on convection, which restricts temperature control. Consequently, a reduction in thickness is more prone to result in temperature non-uniformity, especially in the upper region. This disparity affects the propagation rate of the polymerization front and the temperature distribution, increasing the likelihood of temperature peak instability in thinner resin layers. Conversely, when the width decreases, the occurrence of such overheating phenomena is comparatively less likely.

#### 3.5.3. Effect of Initial Resin Temperature on Polymerization Rate

**Figure 15:**
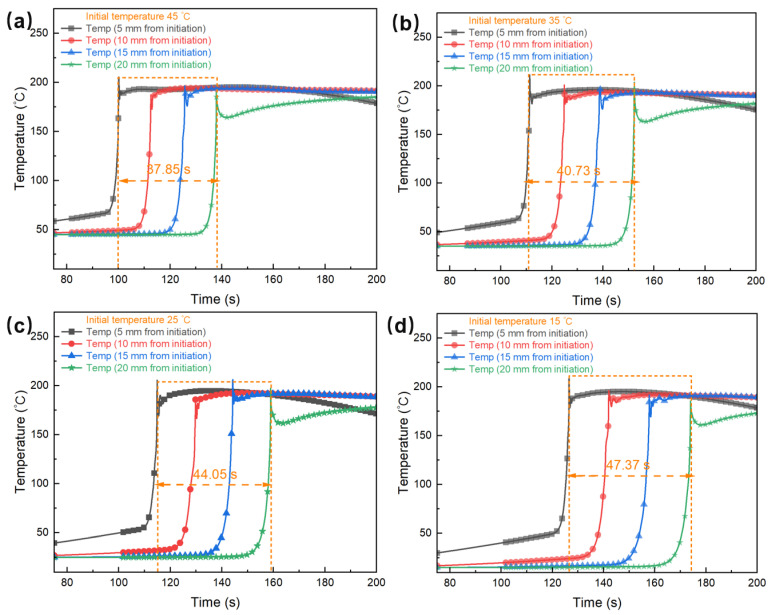
Temperature and degree of cure variation over time for the E51 resin system with different initial temperatures. The initial resin temperatures for a–d are 45 (**a**), 35 (**b**), 25 (**c**), and 15 (**d**) degrees Celsius, respectively. The orange dashed lines represent the time intervals between the first monitoring point and the fourth monitoring point.

Figure 15 shows the simulation results of frontal polymerization under different initial resin temperature conditions. It can be observed that the initial temperature of the resin significantly affects the polymerization reaction rate. When the initial temperature increases, the time interval for the advancement of the polymerization front gradually shortens, indicating an increase in the polymerization rate. This phenomenon occurs because a higher initial resin temperature intensifies molecular thermal motion, effectively lowering the activation energy required for the reaction. Consequently, both the initiation and propagation of the polymerization reaction proceed more rapidly, resulting in an elevated polymerization rate. In contrast, a lower initial temperature results in insufficient energy for the polymerization reaction, which hampers the continuity of the frontal polymerization and the completeness of the reaction. Thus, selecting an appropriate initial temperature for the resin and balancing the reaction rate with temperature control is crucial for optimizing the frontal polymerization process and achieving high-quality curing.

#### 3.5.4. Effect of Resin Heat Transfer Environment on Polymerization Rate

By adjusting the boundary heat transfer conditions of the model, heat transfer through both convection and radiation mechanisms is applied. The governing equations for these two mechanisms are detailed in Equations (7) and (8).

*Q_C_* = *h*·*A_h_*·(*T*_1_ − *T*_0_) (7)

where *Q_C_* is the convective heat transfer, measured in watts (W). *h* is the convective heat transfer coefficient, measured in W·m^−2^·K^−1^, representing the heat transfer capability in the convective process. The specific value of *h* depends on fluid properties, flow conditions, etc., and in this simulation, it is taken as 5 W·m^−2^·K^−1^ [35]. *A_h_* is the heat transfer surface area, measured in square meters (m^2^). *T*_1_ is the surface temperature of the object, measured in degrees Celsius (°C) or Kelvin (K). *T*_0_ is the ambient temperature, measured in degrees Celsius (°C) or Kelvin (K).

*Q_R_* = *σ*·*ϵ*·*A_r_*·(*T*_1_^4^ − *T*_2_^4^) (8)

where *Q_R_* is the radiative heat transfer, measured in watts (W). *σ* is the Stefan–Boltzmann constant, with a value of 5.67 × 10^−8^  W·m^−2^·K^−1^. *ϵ* is the emissivity of the surface of the studied object, ranging from 0 to 1, representing the thermal radiation emission ability of the surface. In this simulation, it is taken as 0.8 [36]. *A_r_* is the radiative surface area, measured in square meters (m^2^). *T*_2_ is the temperature of the surrounding environment or the object receiving the radiation, measured in Kelvin (K).

**Figure 16:**
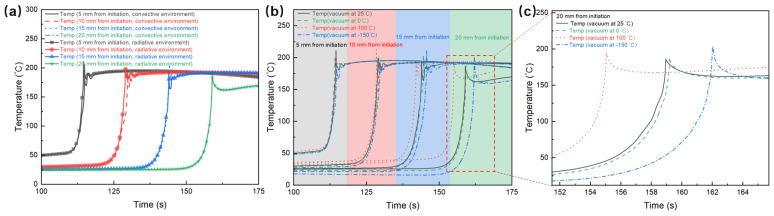
(**a**) Simulated temperature variation over time for the E51 resin system at different points under thermal convection and radiation conditions. (**b**,**c**) Temperature variation curves of the frontal polymerization system under different temperatures in vacuum environment.

The environment temperature is maintained at 25 °C, using a silicone rubber mold with the dimensions of the resin set at 40 mm in width and 12 mm in thickness. The temperature variation curves under both environmental conditions are shown in Figure 16a. When the environment temperature is 25 °C, the temperature variation trends in both conditions are remarkably similar, with close peak temperatures. This observation indicates that under the substantial exothermic conditions in the frontal polymerization reaction zone, the differences in the effects of heat convection and radiation on the polymerization reaction are not significant. In both environments, stable polymerization characteristics are achievable.

In addition, to simulate the polymerization process of resin in the space environment, the heat radiation boundary conditions (vacuum environment) were established, and four environmental temperatures were selected, including 0 °C, 25 °C, 100 °C and −150 °C. The temperature variation curves of each monitoring point are shown in Figure 16b,c. From the figures, it can be observed that the frontal polymerization process can proceed smoothly within the in-orbit temperature range from −150 °C to 100 °C. This indicates that the system has good adaptability in a wide spectrum of in-orbit environmental temperatures. Furthermore, Figure 16c highlights the temperature variations at the final monitoring point after the reaction starts, providing a clearer perspective of the thermal dynamics. At higher environmental temperatures, such as 100 °C, the temperature rise occurs earlier, indicating that higher in-orbit temperatures enhance the polymerization reaction rate, thereby facilitating quicker resin curing and formation. Therefore, frontal polymerization exhibits considerable potential for in-orbit manufacturing, with a certain degree of flexibility in temperature control.

## 4. Conclusions

This work focuses on the reaction mechanism and influencing factors of frontal polymerization for a self-designed epoxy resin system. A comprehensive study was conducted to identify the key factors that affect the frontal polymerization rate and curing characteristics of the epoxy resins under the RICFP mechanism. The main conclusions are detailed below.

The synergistic mechanism of RICFP by PAG and RTI within the E51 resin system was clarified. PAG generates superacid to initiate the ring opening of epoxy groups, while RTI releases free radicals to further enhance the initiation efficiency. Based on DSC tests at different heating rates, a self-catalytic kinetic model of the E51 system was obtained using the Kissinger method. The apparent activation energy was determined to be 124 kJ·mol^−1^. The fitting results of the self-catalytic model indicate that this system possesses a high reaction initiation energy threshold and rapid reaction characteristics.

The effects of initiator ratio and the introduction of alicyclic epoxy resins on the frontal polymerization behavior and the properties of the curing process were investigated. The increase in PAG concentration significantly accelerates the polymerization rate and raises the peak temperature, while the variation in RTI concentration has less impact. The alicyclic resins, specifically E221 and EGA90, demonstrate distinct effects on frontal polymerization behavior. The addition of E221 notably enhances the polymerization rate, while EGA90 is more effective in increasing the heat release. Furthermore, due to the inherent rigid structure of the alicyclic resins, their introduction leads to an increase in the stored energy modulus of the cured product, while the glass transition temperature decreases. This phenomenon is attributed to the formation of a heterogeneous crosslinking network resulting from the introduction of alicyclic resins.

A finite element simulation of the curing and heat transfer processes was performed to analyze the impact of various factors, including mold material, cross-sectional area and initial temperature, on the polymerization behavior of the E51 resin system. The use of silicone rubber mold promotes a more uniform temperature distribution, whereas steel mold, due to faster heat dissipation, reduces the polymerization rate. Increasing the cross-sectional area enhances the ability to accumulate heat, thereby accelerating the polymerization process. Moreover, a higher initial temperature of the resin significantly lowers the energy required to initiate the reaction, thus enhancing the polymerization rate. Additionally, under a vacuum environment simulated at temperatures ranging from −150 °C to 100 °C, the E51 resin system successfully completes the reaction, indicating its adaptability for in-orbit manufacturing.

This study advances the understanding of epoxy resin FP in several points. Firstly, unlike prior works focusing on BF_3_-amine complexes or ROMP systems, we developed a RICFP mechanism using a hybrid initiator system (PAG + RTI). This approach combines the stability of a thermal initiator with the rapid activation of a photoacid generator, enabling precise control over reaction kinetics and avoiding hygroscopicity problems inherent to BF_3_-based systems. Secondly, the established kinetic model explicitly links resin architecture to polymerization rates through molecular-level analysis. Thirdly, the integration of finite element simulation with experimental validation provides a predictive framework for optimizing FP under space-relevant conditions, especially considering anisotropic heat transfer and vacuum effects. Finally, it demonstrates that adjusting the ratio of RTI to resin regulates the exothermicity and front velocity without requiring inhibitors, which is a significant improvement over DCPD-based FP systems. These advancements collectively enhance the feasibility of FP for large-scale in-orbit manufacturing by balancing reactivity, stability and process scalability.

It needs to be noticed that there are several limitations in this study. Firstly, the experimental setup was conducted under idealized laboratory conditions (e.g., controlled ambient pressure and gravity), which might not replicate the thermal and mechanical challenges of space environments. In addition, the study only focused on short-term properties of the cured resins. Long-term stability of the cured resins under space radiation (e.g., UV, cosmic rays) remains unverified.

Some future works can be considered as follows:

- Validation in simulated space environments, including vacuum chambers and microgravity platforms, to assess FP performance under operational constraints.
- Integration of anisotropic material properties (e.g., fiber-reinforced composites) into the simulation framework.
- Investigation of radiation resistance through accelerated aging tests, enabling the development of radiation-tolerant epoxy formulations.
- Exploration of hybrid initiator systems, such as the one combining RTIs with latent catalysts to further enhance process control and storage stability.

Addressing these issues will advance the scalability and reliability of FP for in-orbit manufacturing, bridging the gap between laboratory research and industrial application.

## Figures and Tables

**Table 1:** Physical and thermal properties of different materials used in finite element simulation.

Material	Density (g·cm^−3^)	Temperature (K)	Specific Heat Capacity (J·g^−1^·K^−1^)	Thermal Conductivity (W·m^−1^·K^−1^)
Epoxy resin	1.20	298	1.353	0.226
		433	2.204	0.263
Silicone rubber [23]	1.450	\	1.20	0.27
Steel	8.03	\	0.502	16.27

**Table 2:** Fitting results of self-catalyzed curing kinetics model parameters for the E51 system at different heating rates.

*β* (K·min^−1^)	*A* (s^−1^)	*m*	*n*	Coefficient of Determination
5	3.67383 × 10^14^	0.52713	2.93615	0.99068
10	3.12051 × 10^14^	0.45970	2.90435	0.99510
15	4.79796 × 10^14^	0.62179	3.51995	0.99262
20	3.41255 × 10^14^	0.51286	3.06824	0.99754
25	2.96070 × 10^14^	0.57014	3.08988	0.99711
Average	3.59311 × 10^14^	0.53832	3.10371	/

**Table 3:** Polymerization rate and peak temperature of the E51 system at different RTI concentrations.

PAG:RTI (wt.%:wt.%)	Velocity of FP (cm·min^−1^)	Temperature_max_ (℃)
1:0.5	2.06	218.7
1:1	1.98	219.4
1:1.5	2.05	211.9
1:2	2.12	214.2

## Data Availability

The original contributions presented in this study are included in the article. Further inquiries can be directed to the corresponding author.

## References

[1] Wang G., Zhao W., Liu Y., Cheng T. (2004). Review of space manufacturing technique and developments. Sci. China-Phys. Mech. Astron..

[2] Sacco E., Moon S.K. (2019). Additive manufacturing for space: Status and promises. Int. J. Adv. Manuf. Technol..

[3] Zhang J., Wang G.J., Chen X.Y., Li X.J. (2024). 3D Printing applications for large-scale construction in space. Space Electron. Technol..

[4] Bourseau F., Grugeon S., Lafont U., Dupont L. (2023). 3D printing of solid polymer electrolytes by fused filament fabrication: Challenges towards in-space manufacturing. J. Phys. Energy.

[5] Coughlin N., Drake B., Fjerstad M., Schuster E., Waege T., Weerakkody A., Letcher T. (2019). Development and mechanical properties of basalt fiber-reinforced acrylonitrile butadiene styrene for in-space manufacturing applications. J. Compos. Sci..

[6] Kaligar A.B., Kumar H.A., Ali A., Abuzaid W., Egilmez M., Alkhader M., Abed F., Alnaser A.S. (2022). Femtosecond laser-based additive manufacturing: Current status and perspectives. Quantum Beam Sci..

[7] Hoyt R.P. SpiderFab: An architecture for self-fabricating space systems. Proceedings of the AIAA Space 2013 conference and exposition.

[8] Kantaros A., Ganetsos T., Petrescu F.I.T. (2024). Transforming object design and creation: Biomaterials and contemporary manufacturing leading the way. Biomimetics.

[9] Suslick B.A., Hemmer J., Groce B.R., Stawiasz K.J., Geubelle P.H., Malucelli G., Mariani A., Moore J.S., Pojman J.A., Sottos N.R. (2023). Frontal polymerizations: From chemical perspectives to macroscopic properties and applications. Chem. Rev..

[10] Gao Y., Shaon F., Kumar A., Bynum S., Gary D., Sharp D., Pojman J.A., Geubelle P.H. (2021). Rapid frontal polymerization achieved with thermally conductive metal strips. Chaos.

[11] Centellas P., Yourdkhani M., Vyas S., Koohbor B., Geubelle P., Sottos N. (2022). Rapid multiple-front polymerization of fiber-reinforced polymer composites. Compos. Part A-Appl. Sci. Manuf..

[12] Chechilo N.M., Khvilivitskii R.J., Enikolopyan N.S. (1972). On the phenomenon of polymerization reaction spreading. Dokl. Akad. Nauk SSSR.

[13] Luo T., Ma Y., Cui X. (2024). Review on Frontal Polymerization Behavior for Thermosetting Resins: Materials, Modeling and Application. Polymers.

[14] Robertson I.D., Yourdkhani M., Centellas P.J., Aw J.E., Ivanoff D.G., Goli E., Lloyd E.M., Dean L.M., Sottos N.R., Geubelle P.H. (2018). Rapid energy-efficient manufacturing of polymers and composites via frontal polymerization. Nature.

[15] Robertson I.D., Dean L.M., Rudebusch G.E., Sottos N.R., White S.R., Moore J.S. (2017). Alkyl phosphite inhibitors for frontal ring-opening metathesis polymerization greatly increase pot life. ACS Macro Lett..

[16] Li F., Liu Y., Leng J. (2020). Progress of shape memory polymers and their composites in aerospace applications. J. Astronaut..

[17] An Y., Jang J.H., Youk J.H., Yu W.-R. (2021). Frontally polymerizable shape memory polymer for 3D printing of free-standing structures. Smart Mater. Struct..

[18] Scognamillo S., Bounds C., Thakuri S., Mariani A., Wu Q., Pojman J.A. (2014). Frontal cationic curing of epoxy resins in the presence of defoaming or expanding compounds. J. Appl. Polym. Sci..

[19] Zhou J., Jia S., Fu W., Liu Z., Tan Z. (2016). Fast curing of thick components of epoxy via modified UV-triggered frontal polymerization propagating horizontally. Mater. Lett..

[20] Mariani A., Bidali S., Fiori S., Sangermano M., Malucelli G., Bongiovanni R., Priola A. (2004). UV-ignited frontal polymerization of an epoxy resin. J. Polym. Sci. Part A Polym. Chem..

[21] Bomze D., Knaack P., Liska R. (2015). Successful radical induced cationic frontal polymerization of epoxy-based monomers by C–C labile compounds. Polym. Chem..

[22] Groce B.R., Gary D.P., Cantrell J.K., Pojman J.A. (2021). Front velocity dependence on vinyl ether and initiator concentration in radical-induced cationic frontal polymerization of epoxies. J. Polym. Sci..

[23] Staal J., Smit E., Caglar B., Michaud V. (2023). Thermal management in radical induced cationic frontal polymerisation for optimised processing of fibre reinforced polymers. Compos. Sci. Technol..

[24] Zhang J., Liang B., He X., Yi M., Yang W., Hu J., Zeng K., Yang G. (2024). Curing kinetics and thermal properties of [2,2] paracyclophane/bisphthalonitrile-terminated imide resins. Mater. Today Commun..

[25] Zeng Z., Zhao T., Gong Y., Yu Z. (2024). Synthesis, curing kinetics and properties of novel aromatic multifunctional epoxy resin. Eur. Polym. J..

[26] Zhao H., Xu S., Guo A., Li J., Liu D. (2021). The curing kinetics analysis of four epoxy resins using a diamine terminated polyether as curing agent. Thermochim. Acta.

[27] Bao C., Wang Y., Mushtaq R.T., Zhang K., Li X., Chen X. (2022). Preparation, characterization, and curing kinetics of elevated and cryogenic temperature-resistant epoxy resin composites. Polym. Test..

[28] Li J., Zhao H., Sui G. (2022). Renewable green reactive diluent for bisphenol a epoxy resin system: Curing kinetics and properties. RSC Adv..

[29] Zhu J., Xia Y., Liu L., Yan S., Zeng Y., Zhang R., Zhang X., Sheng Y. (2024). Comparative study of the kinetic behaviors and properties of aromatic and aliphatic bismaleimides. Thermochim. Acta.

[30] Wang H., Dayo A.Q., Wang J.-Y., Liu W.-B. (2020). Synthesis, curing kinetics and thermal properties of two novel quinoxaline-based mono-and bismaleimides. Thermochim. Acta.

[31] Zanjanijam A.R., Wang X., Ramezani M., Holberg S., Johnson P.A. (2023). Phenolic resin/coal char composites: Curing kinetics and thermal/mechanical performance. Polymer.

[32] Kessler M.R., White S.R. (2002). Cure kinetics of the ring-opening metathesis polymerization of dicyclopentadiene. J. Polym. Sci. Part A Polym. Chem..

[33] Malik M.S., Wolfahrt M., Sangermano M., Schlögl S. (2022). Effect of a Dicycloaliphatic Epoxide on the Thermo-Mechanical Properties of Alkyl, Aryl Epoxide Monomers Cured via UV-Induced Cationic Frontal Polymerization. Macromol. Mater. Eng..

[34] dell’Erba I.E., Arenas G.F., Schroeder W.F. (2016). Visible-light photopolymerization of DGEBA promoted by silsesquioxanes functionalized with cycloaliphatic epoxy groups. Polymer.

[35] Khalifa A.-J.N. (2001). Natural convective heat transfer coefficient–a review: I. Isolated vertical and horizontal surfaces. Energy Convers. Manag..

[36] Snyder W.C., Wan Z., Zhang Y., Feng Y.-Z. (1998). Classification-based emissivity for land surface temperature measurement from space. Int. J. Remote Sens..

